# Out-of-hospital rescue medication in dogs with emergency seizure disorders: an owner perspective

**DOI:** 10.3389/fvets.2023.1278618

**Published:** 2023-10-02

**Authors:** Charlotte Kähn, Sofie F. M. Bhatti, Sebastian Meller, Nina Meyerhoff, Holger A. Volk, Marios Charalambous

**Affiliations:** ^1^Department of Small Animal Medicine and Surgery, University of Veterinary Medicine Hannover, Hannover, Germany; ^2^Small Animal Department, Ghent University, Ghent, Belgium

**Keywords:** epilepsy, status epilepticus (SE), cluster seizures (CS), management, canine (dog), home

## Abstract

**Background:**

Emergency seizure disorders such as status epilepticus and cluster seizures are unlikely to cease spontaneously while prolonged seizure activity become progressively more resistant to treatment. Early administration of rescue medication in canine epileptic patients, in particular benzodiazepines, at seizure onset by the owners can be life-saving and brain protecting. Clinical studies in dogs evaluating the use of rescue medication in hospital environment exist, however, the owner perspective has not been assessed to date.

**Hypothesis or objectives:**

To evaluate the use of rescue medication in dogs with seizure emergencies by the owner at home.

**Method:**

Observational study based on online surveys of owners of dogs with emergency seizure disorders.

**Results:**

The questionnaire was answered by 1,563 dog owners, of which 761 provided complete and accurate answers suitable for analysis. Of these, 71% administered diazepam, 19% midazolam, 6% levetiracetam, 3% lorazepam, and 4% more than one rescue or other medication. Overall, the success rates based on owners’ perspective for intranasal midazolam and rectal diazepam were 97 and 63%, respectively. Owners reported a compliance level of 95 and 66% for intranasal midazolam and rectal diazepam administration, respectively.

**Conclusions and clinical importance:**

Even though rectal diazepam was the most used rescue medication in this survey population, intranasal midazolam was perceived by the owners as a better option regarding effectiveness, time to seizure cessation and owner compliance.

## 1. Introduction

Emergency seizure disorders, including status epilepticus (SE) and cluster seizures (CS), are commonly presented in both primary and specialty practices. SE is defined as a seizure lasting ≥5 min or ≥ 2 seizures with incomplete recovery in between seizures. SE can occur in dogs with reactive seizures, idiopathic epilepsy, or structural epilepsy (1, 2). CS are defined as two or more self-limiting seizures occurring within a 24-h period (1, 3). Approximately, 0.5–2.6% of the dogs presented to hospital manifest SE. Of the dogs admitted to hospital for seizures, 16.5% have SE; 58% of these dogs show SE as the first clinical manifestation of a seizure disorder. Overall, 20–60% of dogs with idiopathic epilepsy experience at least one SE. The overall mortality for SE has been reported to be 25.3–38.5%. (2, 4).

SE can be life-threatening and requires immediate care to avoid permanent brain damage (e.g., excitotoxicity, neuronal cell necrosis) or systemic dysfunction (e.g., shock, cardiorespiratory collapse, electrolyte disturbances, acidosis). The complications arising from SE are proportionally related to the duration of seizure activity (2, 5, 6). SE and CS often occur at home (7). Clinicians and especially owners should adopt a rapid and effective action plan for ceasing seizure emergencies before reaching refractory stages or occurrence of permanent brain damage; ideally seizures should terminate before reaching 30 min of continuous seizure activity (2, 5, 6, 8). The most common and first-line rescue medications used to manage seizure emergencies at home are benzodiazepines (BZDs), mainly diazepam (DZP) and midazolam (MDZ). These are potent and effective medications which can be administered in non-intravenous (IV) routes (2). Pharmacokinetic studies have demonstrated that intranasal administration of DZP, MDZ, triazolam, and flurazepam rapidly reaches maximal serum concentrations in dogs (8–11). Various studies have shown that intranasal MDZ has a high efficacy. In a meta-analysis in human medicine, which compared sublingual lorazepam, intranasal lorazepam, buccal MDZ, intranasal MDZ, and rectal DZP, showed that intranasal MDZ has the highest efficacy, followed by buccal MDZ and rectal DZP (12). Another study in 1991 has shown that the time between administration of intranasal MDZ to reach the highest plasma concentration is two times shorter and the plasma concentration is 2.9 times higher than oral administration (9). The therapeutic serum concentration of MDZ has not yet been determined in dogs, but in humans is 0.04 μg/mL. In dogs, DZP therapeutic concentration has been reported, which ranges from 0.15 to 0.5 μg/mL serum in dogs. In addition, MDZ is likely 5–6 times more potent than DZP (2, 13).

A study in humans showed that the first-line treatment is commonly delayed in both out-of-hospital and in-hospital settings. Rescue medications were administered only in 37.5% of patients at home (14) and even with prior diagnosis of epilepsy, rescue medication was administered only to a low number of patients from their family members (14). In another study, patients who received a delayed first benzodiazepine, i.e., > 10 min, had a higher mortality rate and a higher probability for receiving further multiple antiseizure medications (ASMs) than only BZDs (15). Therefore, it is by far vital to administer BZDs early in the course of the emergency seizures, i.e., at home, to prevent high rates of complications and mortality as well as refractory stages. In veterinary medicine, there are two clinical studies investigating the clinical efficacy of BZDs in dogs, in particular intranasal MDZ, as a rescue medication for SE within hospital settings (16, 17), however, no data exists regarding the evaluation of the emergency seizure treatment options at home by the owners. The aim of this online survey was to assess the use of rescue medication used at home from an owner perspective and identify any gaps that may lead to inadequate or delayed treatment of seizure emergencies.

## 2. Materials and methods

An observational study was designed based on online surveys of owners of dogs suffering from seizure disorders. The survey’s questions were developed using veterinary expertise and previously published questionnaires in the pediatric setting (18). The first section consisted of five questions regarding basic information such as dogs’ signalment and general clinical status. The second section included 15 questions regarding the semiology, course, diagnosis, and treatment of an epileptic disorder as well as the quality of life (QoL) of dogs with epilepsy and their owners. The third section included 22 questions assessing the use of rescue medication in emergency seizure disorders at home. The questionnaire was provided *via* the online survey software limesurvey^1^ and distributed *via* social media. The data was collected from September to the end of November 2022. The questionnaire was published after receiving permission from the University’s data protection office. Responses were used for analysis if they were completely answered and information about the rescue medication used was included. Descriptive statistics were used to evaluate the data obtained. The data were analysed using Microsoft Excel.

## 3. Results

### 3.1. Disease characteristics and quality of life

**Figure 1:**
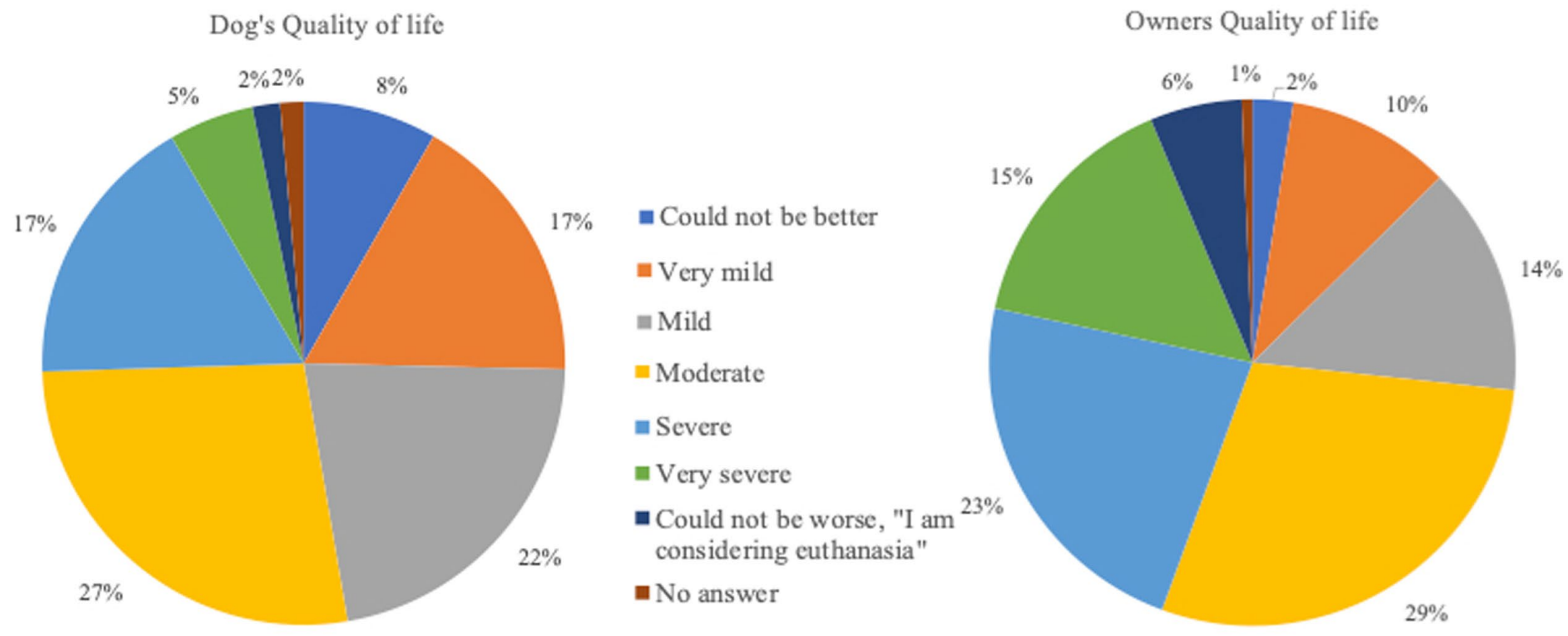
Quality of life of owners and dogs with emergency seizure disorders. (Very mild = very mild decrease of Quality of life; Mild = mild decrease of Quality of life; Moderate = moderate decrease of Quality of life; Severe = severe decrease of Quality of life; Very severe = very severe decrease of Quality of life).

The questionnaire was answered by 1,563 dog owners, of which 761 provided complete responses and were therefore analysed. Table 1 summarizes general information about the study population. Owners from various countries participated with Germany to be the most common, followed by Belgium, UK and then USA. The QoL of both owners and dogs was adversely affected by the seizure disorders (Figure 1).

**Table 1:** General information regarding dogs’ disease characteristics and clinical status.

Country	Germany 54.92% (418/761), Belgium 12.75% (97/761), UK 11.96% (91/761), USA 8.54% (65/761), The Netherlands 3.29% (25/761), Austria 2.23% (14/761), Italy 0.79% (6/761), Other 5.91% (45/761)
Breed	Crossbreed 28.91% (220/761), Border Collie 7.23% (55/761), Labrador Retriever (6.57%) 50/761, Australia Shephard 4.20% (32/761); Beagle 3.42% (26/761), Golden Retriever 3.29% (25/761), Collie 2.76% (21/761), Belgian Shephard 2.63% (20/761), German Shephard 2.37% (18/761), Other 38.63% (294/761)
Sex	Entire-male 25.36% (193/761), male-neutered 39.95% (304/761), entire-female 10.78% (82/761), female-neutered 23.92% (182/761)
Age at the date of the survey	6.35 years (mean age); 6 years (median); 0–19 (range)
Diagnostics	History, signalment and blood/urine tests 91.33% (695/761; Tier I: 93.29%); previous elements plus MRI or CT and CSF 38.37% (292/761; Tier II: 42.03%); previous elements plus EEG 1.05% (8/761; Tier II: 1.04%)
Diagnosis	Idiopathic epilepsy 88.17% (671/761); Structural epilepsy 3.02% (23/761); Reactive seizures 0.92% (7/761); I do not know/no answer 7.88% (60/761)
Clinical manifestation of seizures	Generalised 77.66% (591/761); Generalised with focal onset (100/761) 13.14%; Focal (62/761) 8.15%; I do not know (5/761) 0.66%
Incidence of cluster seizures	Never (107/761) 14.06%; Once per year (72/761) 9.46%; Not monthly but more than once per year (359/761) 47.17%; Once per month (103/761) 13.53%; More than once per month (74/761) 9.72%; I do not know/Not applicable (46/761) 6.04%
Incidence of status epilepticus	Never (298/761) 39.16%; Once per year (60/761) 7.88%; Not monthly but more than once per year (225/761) 29.57%; Once per month (72/761) 9.46%; More than once per month (29/761) 3.81%; I do not know/Not applicable (77/761) 10.12%
Long-term medication	Monotherapy 51.78% (394/761); Multiple drug therapy 48.23% (367/761) Phenobarbital 76.35% (581/761); Potassium bromide 25.36% (193/761); Levetiracetam 23.92% (182/761); Zonisamide 4.60% (35/761); Gabapentin 3.29% (25/761); Pregabalin 1.18% (9/761); Other medication 3.15% (24/761)

### 3.2. Rescue medication

**Figure 2:**
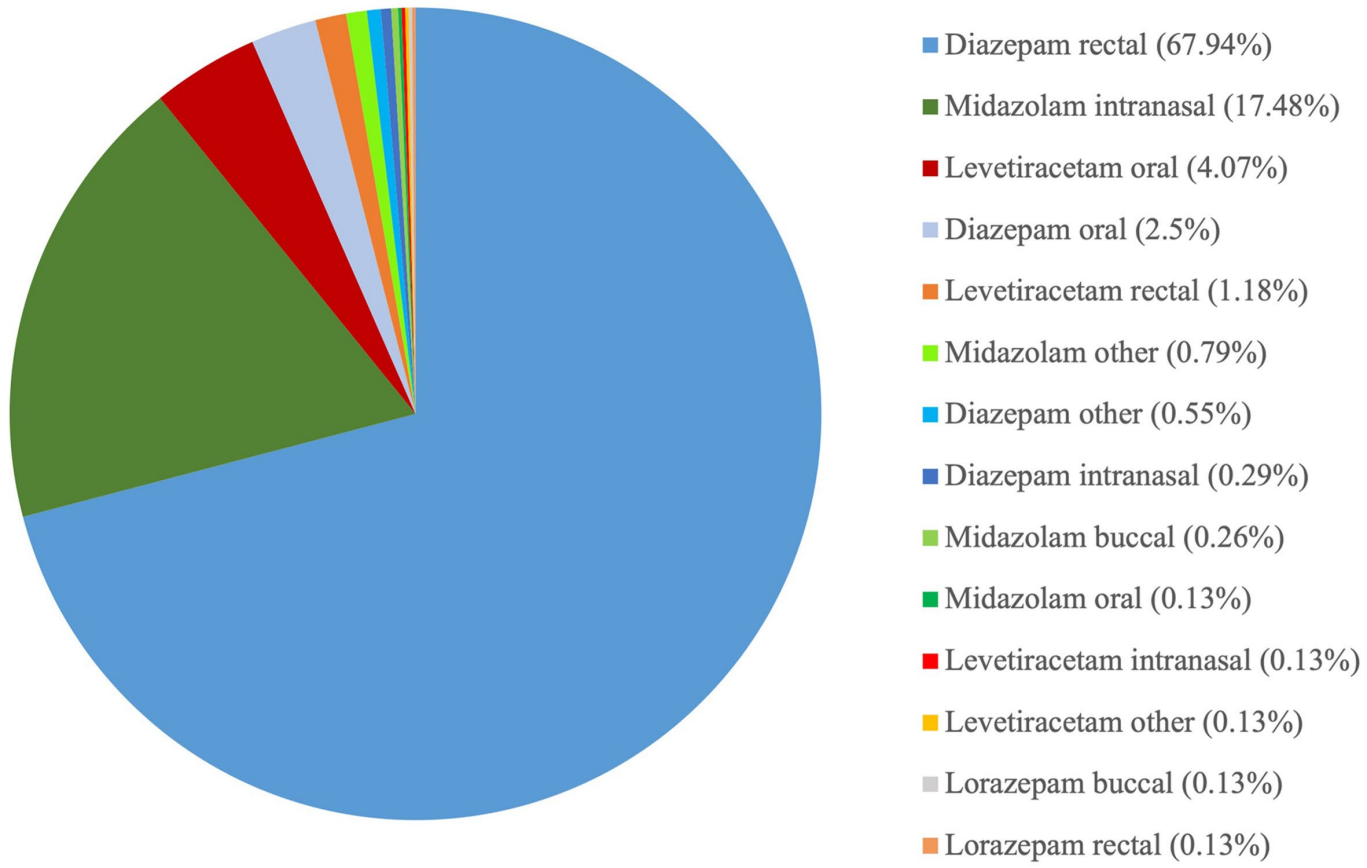
Rescue medication type and administration route used by the owners.

**Figure 3:**
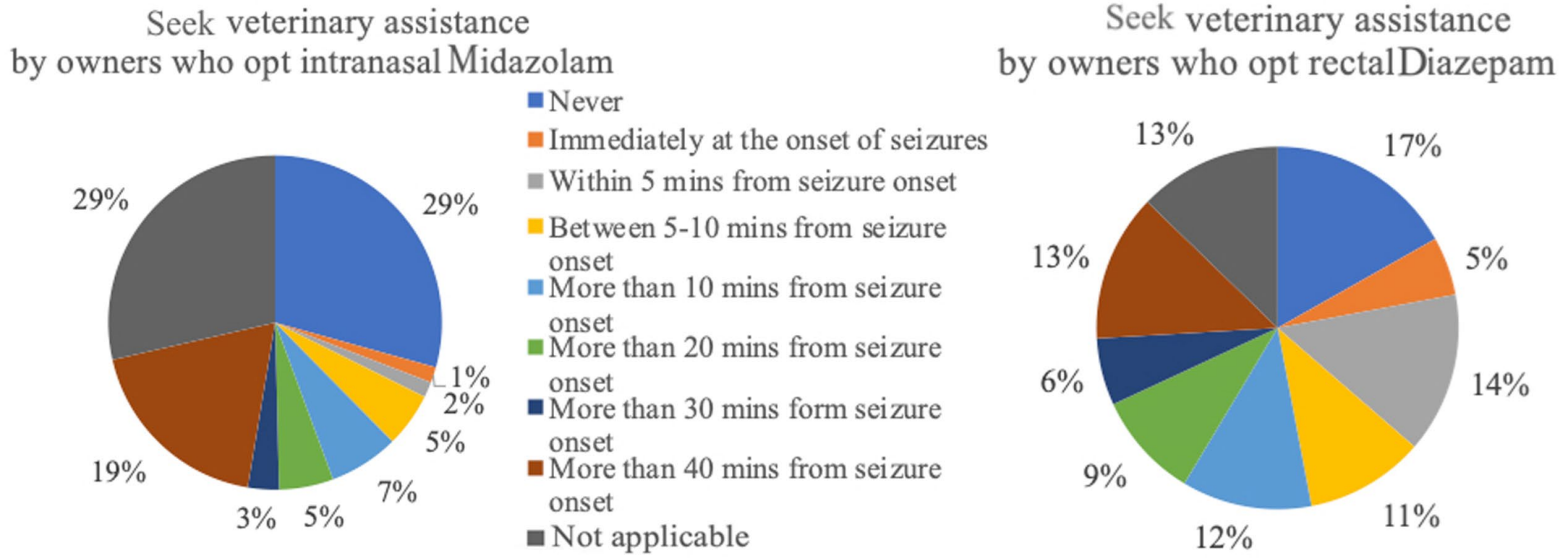
Seek veterinary assistance by owners who opt intranasal Midazolam (*n* = 133) versus rectal Diazepam (*n* = 517).

**Figure 4:**
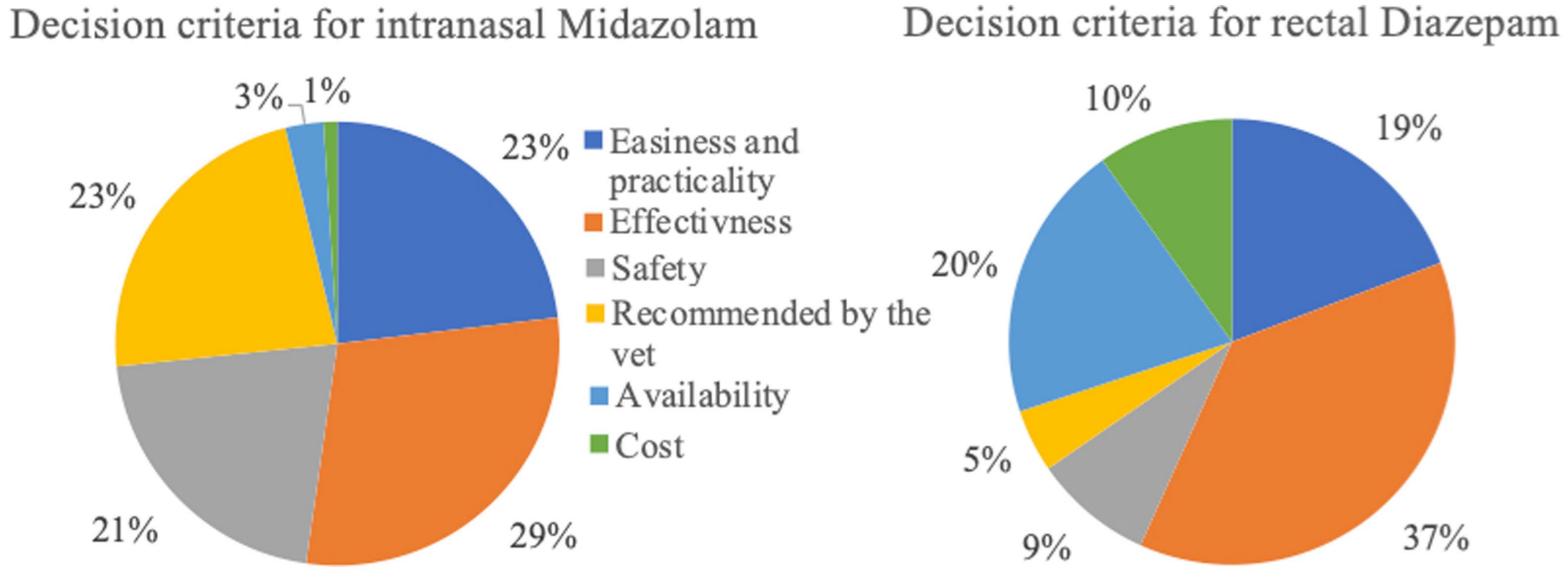
Decision criteria of respondents for selecting intranasal Midazolam (*n* = 133) or rectal Diazepam (*n* = 517).

**Figure 5:**
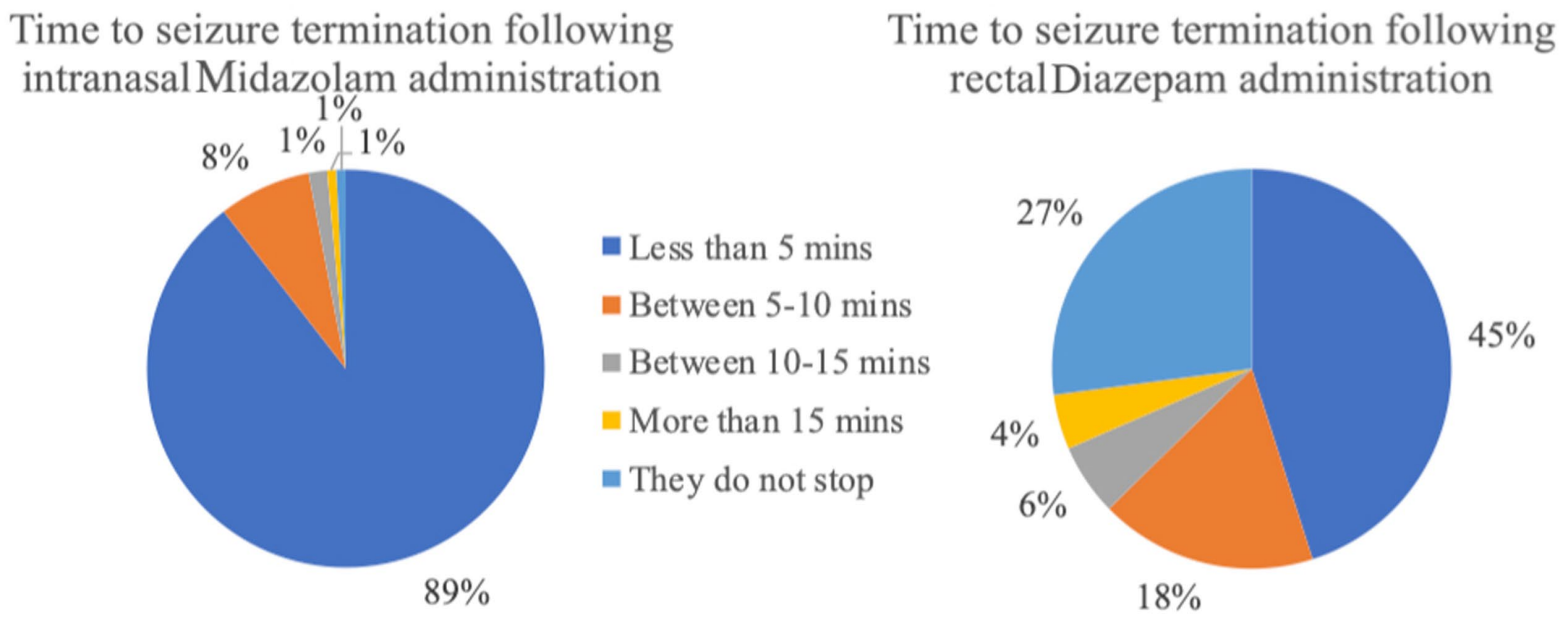
Time to seizure termination following administration of the rescue medication (intranasal Midazolam *n* = 133; rectal Diazepam *n* = 517).

**Figure 6:**
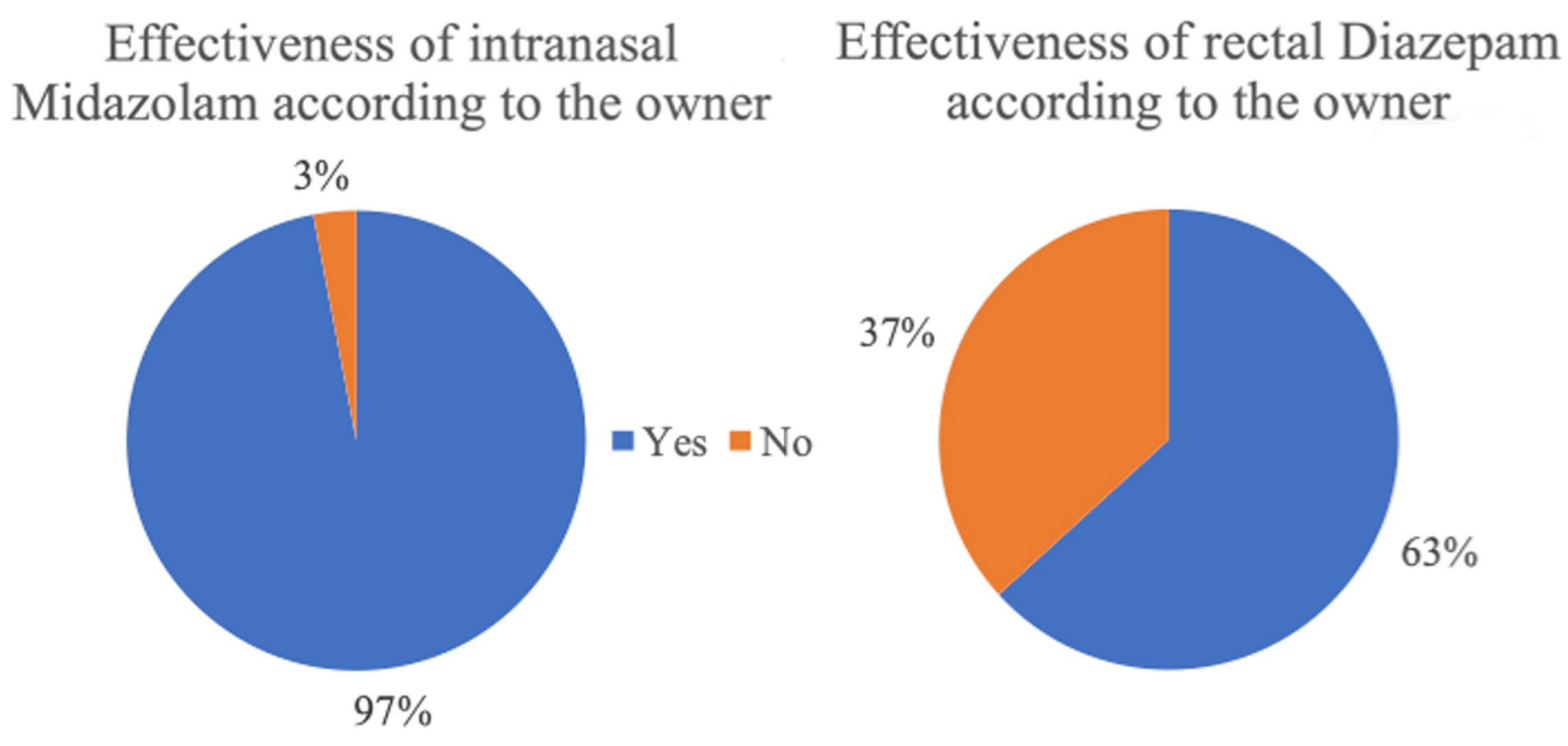
Effectiveness of the rescue medication based on the owner perspective (intranasal Midazolam n = 133; rectal Diazepam *n* = 517).

**Figure 7:**
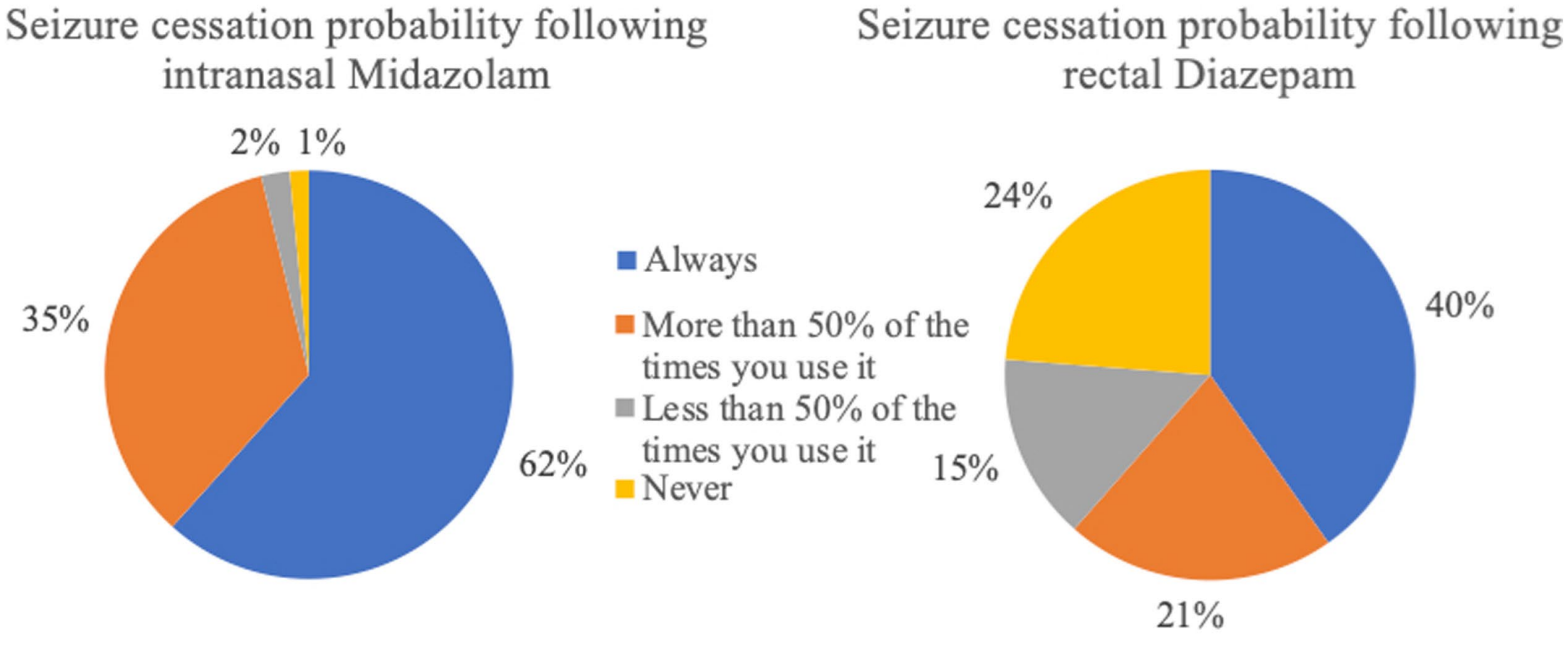
Probability of seizure cessation following administration of the rescue medication (intranasal Midazolam *n* = 133; rectal Diazepam *n* = 517).

**Figure 8:**
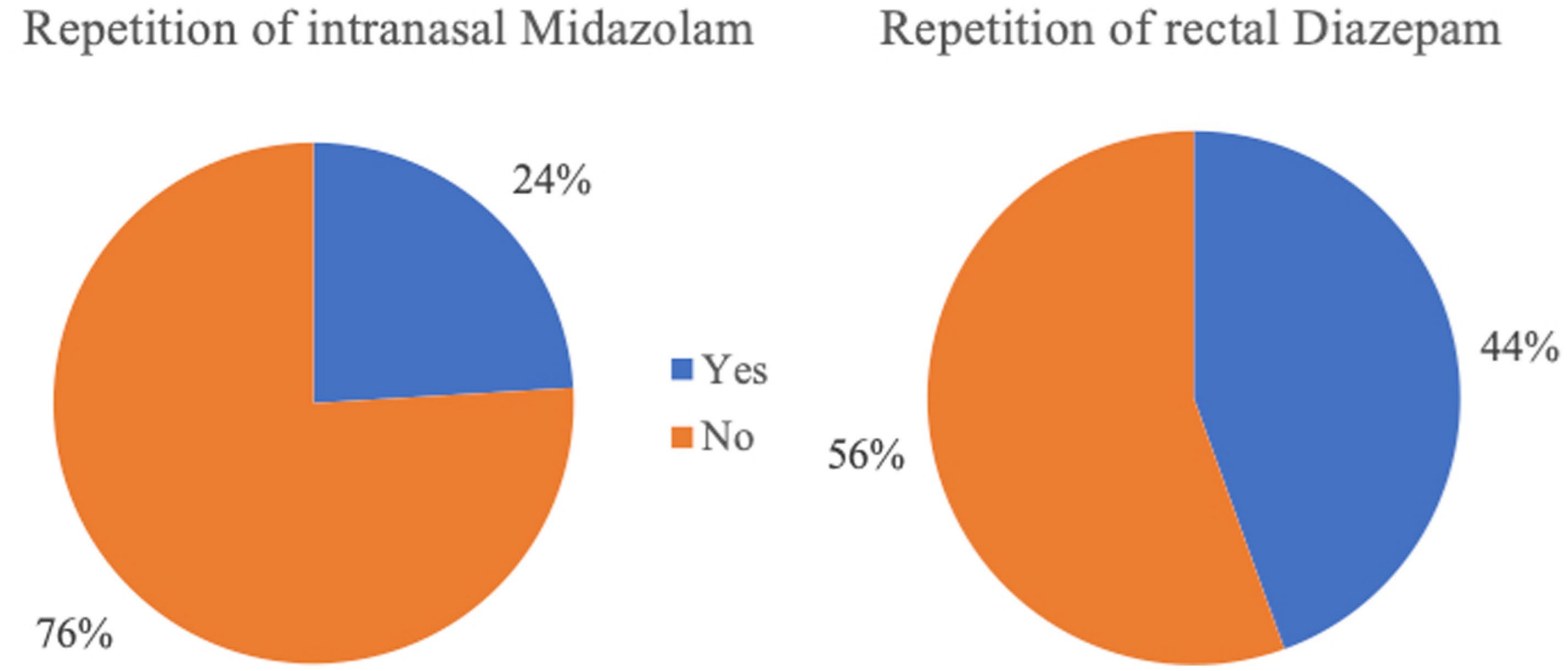
Rates of re-administration of the rescue medication following the initial dosage (intranasal Midazolam *n* = 133; rectal Diazepam *n* = 517).

**Figure 9:**
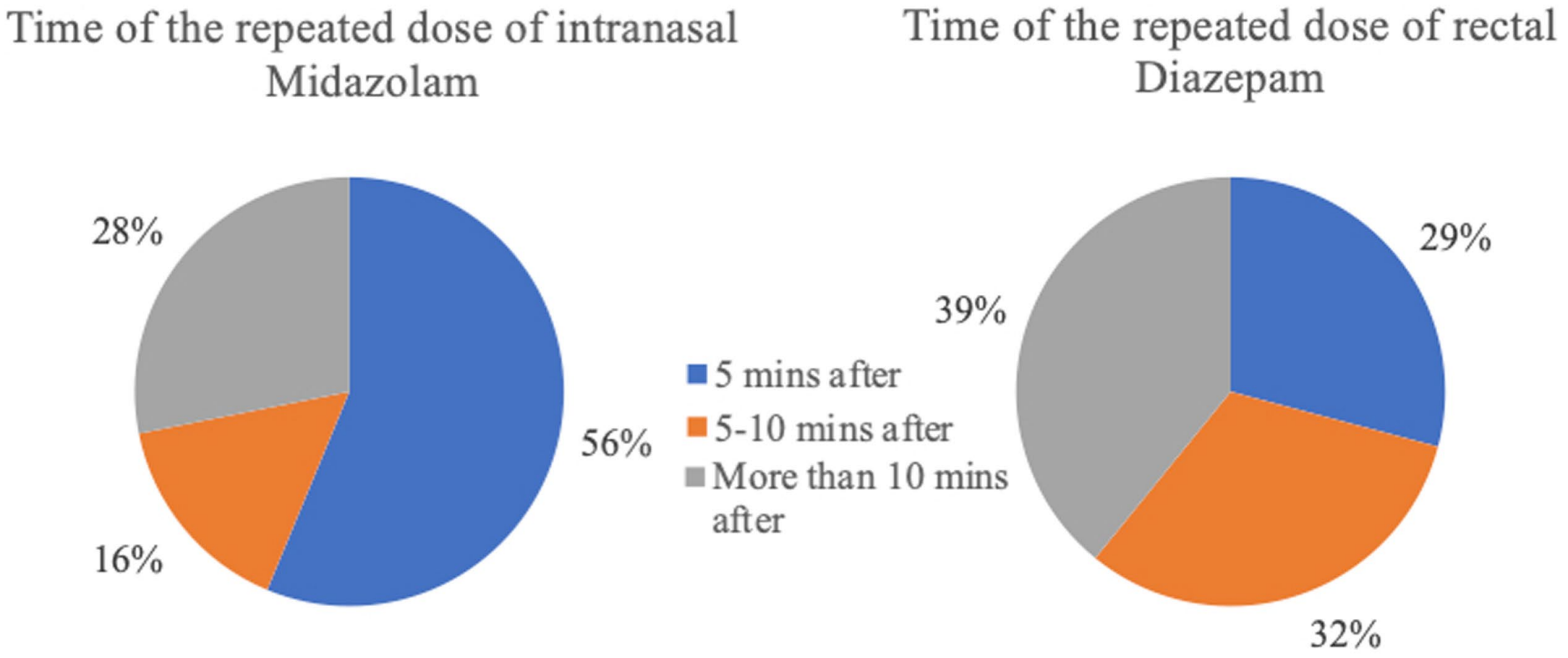
Time of the repeated dosage after administration of the initial rescue medication (intranasal Midazolam *n* = 32; rectal Diazepam *n* = 230).

**Figure 10:**
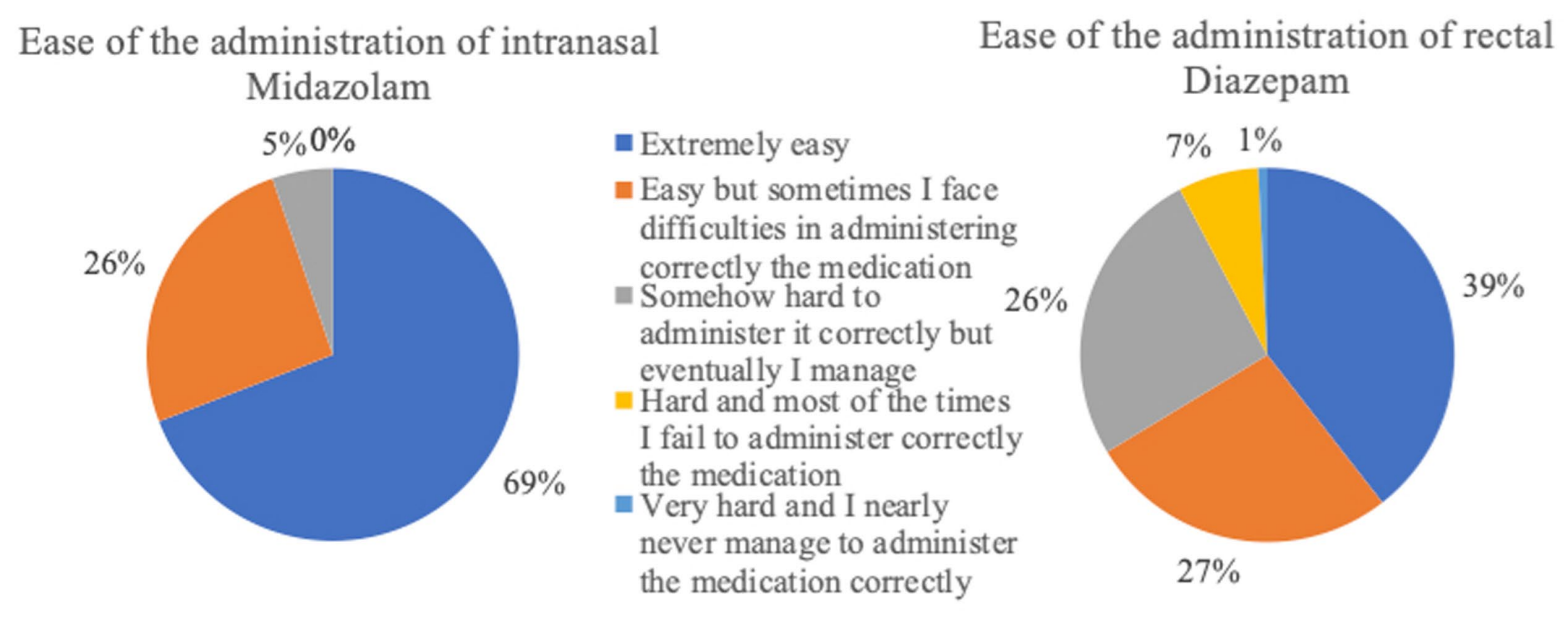
Ease of administration of the rescue medication (intranasal Midazolam *n* = 133; rectal Diazepam *n* = 517).

**Figure 11:**
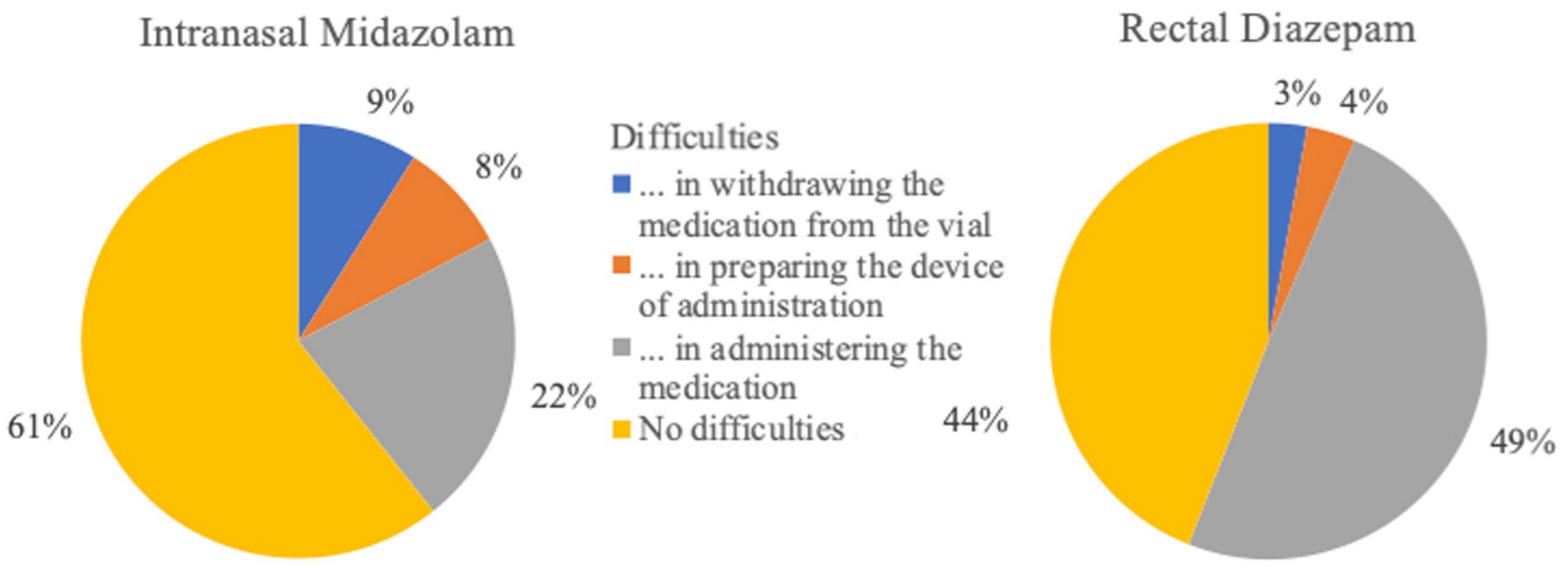
Difficulties in administering and preparing rescue medications (intranasal Midazolam *n* = 133; rectal Diazepam *n* = 517).

**Figure 12:**
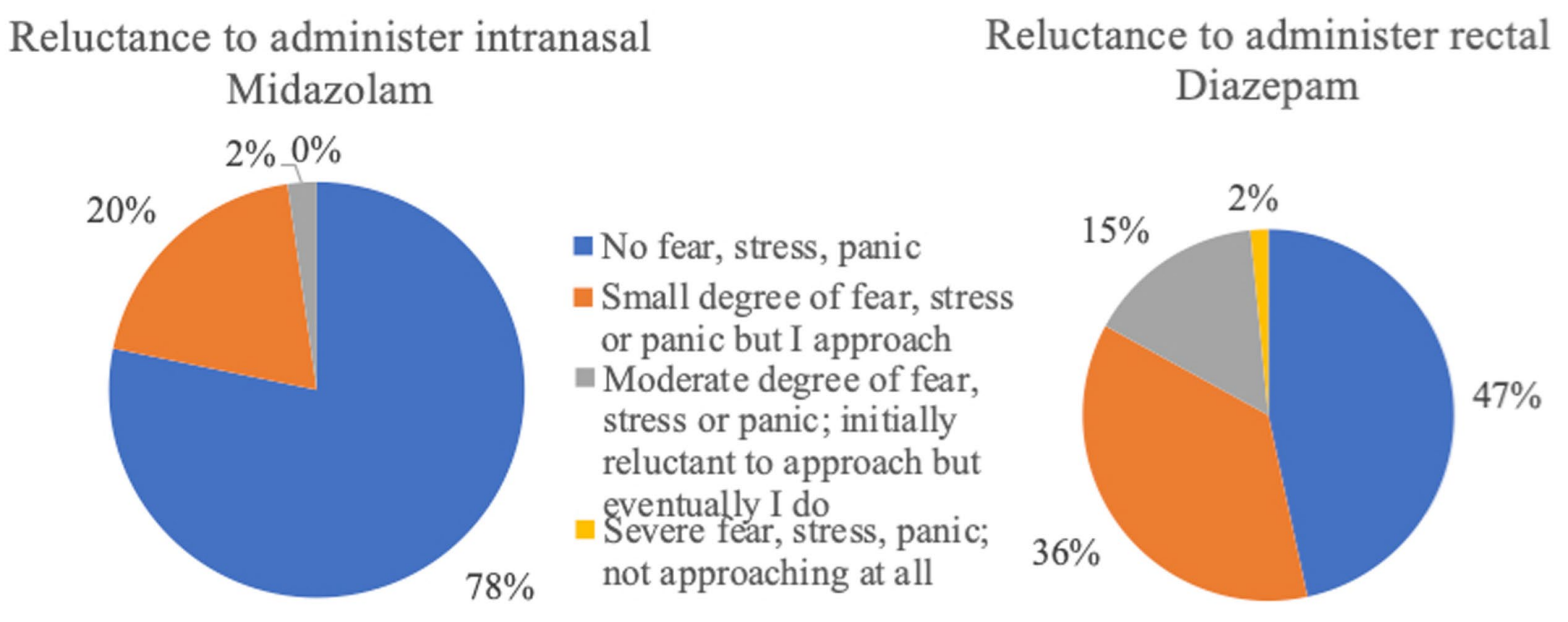
Reluctance to administer rescue medication (intranasal Midazolam *n* = 133; rectal Diazepam *n* = 517).

**Figure 13:**
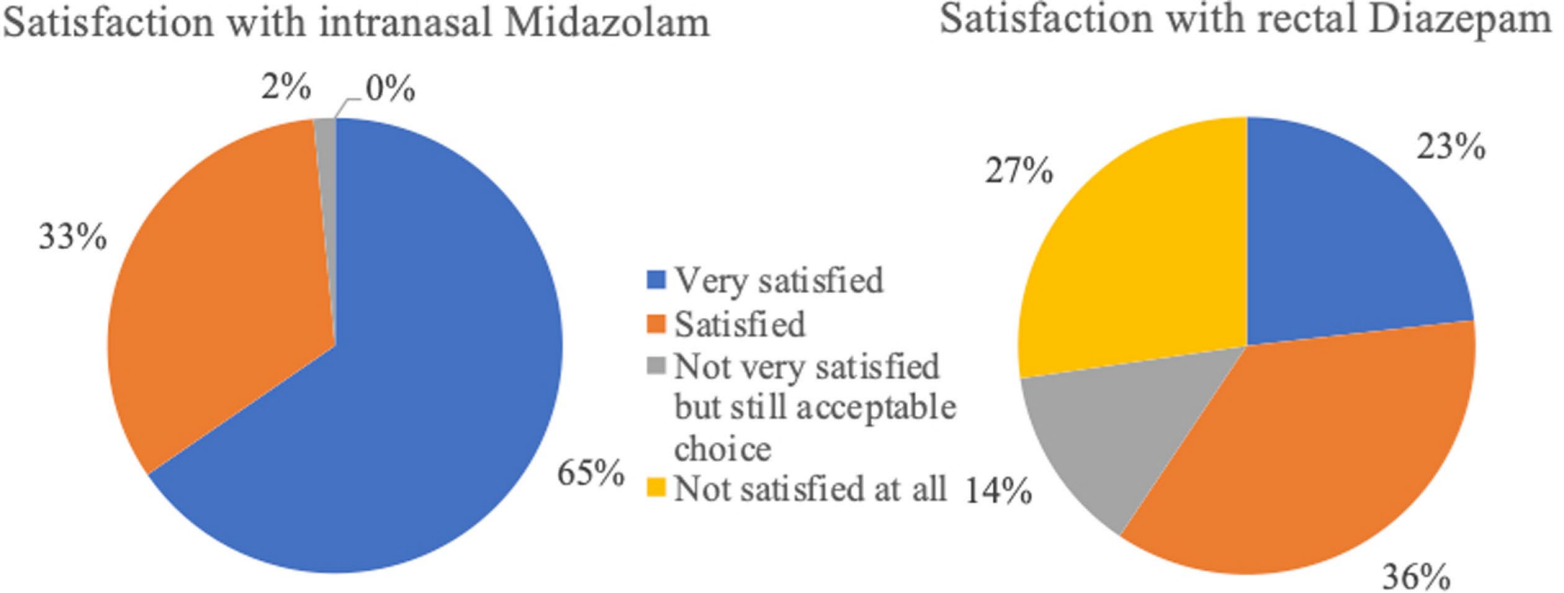
Satisfaction rates following administration of rescue medications (intranasal Midazolam *n* = 133; rectal Diazepam *n* = 517).

Rectal DZP followed by intranasal MDZ were the most common rescue medications used to treat SE at home. Rectal DZP was most commonly used in Germany (79.24%, 332/419), followed by intranasal MDZ (5.49%, 23/419) while intranasal MDZ was the most common rescue medication in Belgium (52.04%, 51/98), followed by rectal DZP (43.88%, 43/98). Overall, based on owners’ perception, intranasal MDZ had higher success as well as owner satisfaction and compliance rates compared to rectal DZP. In addition, in cases where intranasal MDZ was administered, 60.9% of owners bever sought veterinary help, while 33.83, 3, and 2% sought veterinary assistance in <50%, > 50, and 100% of cases, respectively. In cases of rectal DZP, only 42.17% of owners stated that never sought veterinary care, while 22.82, 29, and 6% of owners sought veterinary care in <50%, > 50, and 100% of cases, respectively. Intranasal MDZ was administered *via* a special atomizer/spray device in 88.72% (118/133), a simple syringe (without needle) in 10.53% (14/133), or another delivery method in 0.75% (1/133) of the cases. Rectal DZP was administered *via* a rectal suppository in 38.10% (197/517), a simple syringe (without a needle) in 31.33% (162/517), a rectal tube in 23.6% (122/517), or another delivery method in 4.06% of the cases; in 2.9% (15/517) of the cases, no information regarding the delivery method was provided. Further detailed information regarding the assessment of the rescue medications is provided in Figures 2–13.

## 4. Discussion

To our knowledge, this is the first large-scale owner online survey which not only assesses the use of rescue medication at home, but also underlines the burden of SE on dogs and their caregivers from an owner perspective. Questions in the survey were used to evaluate management of the emergency seizure disorders at home and the communication between veterinary surgeons and owners, the owner compliance, and the impact of SE on the QoL of both dogs and owners.

The results of the survey show that owners consider their own QoL to be remarkable more impaired than the QoL of their dog. Studies in human medicine also highlighted the extent to which the QoL of caregivers is more impacted when compared to the QoL of people suffering from epilepsy (19–21). The care of pets with epilepsy is not as well researched, but there are similarities in the manner between the care of humans and animals with a chronic disorder. One reason for the more impaired QoL of owners is that animals are unconscious during the generalised seizure, hence, the owners are the ones who experience the dramatic manifestation of the ictal stage of seizures. Furthermore, the level of seizure control does not align with the level of involvement of the owners. This lack of correlation can cause owners to feel hopeless, despite their considerable efforts to relieve their dog’s seizures. As a result, overall compliance with epilepsy management may decline (22). In a qualitative study, interviews with owners of dogs with epilepsy showed the impact on owners’ lives (23). For most of them, the unpredictable nature of epilepsy, the timing of the epileptic seizure or even the search for prodromal symptoms were the most severe challenges. Changes in the lifestyle was also a point of consideration. Although many indicated that the change does not necessarily mean a decrease in QoL, alterations in daily planning (2–3 times per day medication), leisure and working schedules (reduction of working hours, giving up work) have adversely affected the lifestyle of the owners. In contrast to all the negative points, the only positive outcome was the development of stronger bond between the animal with epilepsy and the owner (23). Our survey results showed the negative impact that seizure emergencies have on the dogs and owners at emotional, social and financial level as well as emphasized the importance of optimal and effective antiseizure interventions. The emotional level associated with the administration of emergency medication cannot be adequately elucidated in this study. To assess such an emotional impact, personal detailed interviews with owners and application of psychological tests are deemed vital. The survey also provided an insight into the different action plans owners undertake when dealing with SE. Interestingly, the results revealed areas of disparate perceptions among owners living in different countries regarding the management of seizures. Overall, the information in this survey can be used to identify and resolve any gaps requiring more attention during clinician and owner interactions, which may enhance the management of seizure emergencies at home, improve outcomes and ameliorate the QoL of both dogs and owners.

Evidence from multicenter clinical trials show that intranasal administration of MDZ is effective and safe (16, 17) as well as superior to rectal DZP (16) for ceasing SE. Pharmacokinetic studies also showed that intranasal administration of benzodiazepines, including MDZ (8, 9), DZP (10, 11) and flurazepam (9) leads to rapid and efficient absorption by the nasal mucosa and can reach adequate therapeutic serum concentrations quick enough to cease emergency seizures. Intranasal route is characterized by multiple benefits over the rectal route of administration which are thoroughly analysed by Charalambous et al. (2, 16, 17). The results of this survey supported intranasal administration of MDZ as a more satisfactory method of SE management at home, compared to rectal DZP or other interventions at home, from an owner perspective. However, despite the fact that overall clinical or survey-derived evidence shows advantage of intranasal MDZ over rectal DZP, rectal DZP was still the most common rescue medication used at home by the owners. This difference was marked among owners from different countries. According to the study, only 17% of all the respondents used intranasal MDZ. Of these respondents, the largest proportion (38%) were from Belgium. Rectal DZP was used by 68% of all the respondents, with the largest proportion (65%) originating from Germany. Such a finding might indicate either the reluctance of owners to change the traditional methods of treating SE at home or that evidence-based practices are not widely followed in some countries or by specific populations. Another reason why intranasal midazolam is more popular in Belgium is that multiple trials have been primarily led there by Charalambous et al. and owners and veterinarians are more aware of the intranasal administration.

The survey also examined the reasons for owners’ decisions, i.e., whether effectiveness, practicality, recommendations, safety, availability, or cost may influence the choice of specific interventions. Even though this survey reported increased rates of failure with rectal DZP (37%) compared to intranasal MDZ (3%), many owners continue to use rectal DZP. One of the reasons for this could be that owners are not aware of the alternative intranasal administration route for ceasing seizure emergencies at home. The survey found that rectal DZP was chosen mainly for its perceived efficacy, whereas intranasal MDZ was chosen based on its perceived efficacy as well as practicality, safety, and veterinary recommendations. This indicates that many veterinarians already follow evidence-based practices and recommend intranasal MDZ (intranasal MDZ 23%; rectal DZP 5%). A method to widely introduce evidence-based recommendations is the establishment of official guidelines for the treatment of seizure emergencies. Recently an initiative was taken by experts in the field to create official guidelines which can form a common framework for clinicians to follow; these guidelines are expected to be published in late 2023 (Charalambous et al. ACVIM consensus statement on the management of emergency seizure disorders).

Risks such as owners fear of being bitten or causing injury to their dog during administration of intranasal MDZ were not supported in this survey, as more respondents used intranasal MDZ due to its safety than they did for rectal DZP. These risks were evaluated by the clinical trials on intranasal MDZ and were not reported as an issue with intranasal administration by the clinicians (16, 17). Mild difficulties were documented in 45% of cases, which included brief sneezing when administering the medication or difficulty using the device in small nostrils (16). In our survey, 69 and 39% of the respondents who administer intranasal MDZ or rectal DZP, respectively, reported the administration methods as “easy.” Mild, moderate or severe problems were experienced with rectal DZP administration by 61% and with intranasal MDZ by 31%. The most commonly described difficulties in administering rectal diazepam were administration as such by suppository or tube followed by the risk of reverse outflow of the drug from the rectum immediately after application. In the case of intranasal application, sneezing during administration (one report; 0.75%) was a potential risk.

For rectal applications, suppositories or rectal tubes were mostly used; these were types of application that hardly require any preparation. For intranasal application, a mucosal atomization device (MAD) was mostly used. The procedure required opening the capsule, pulling the drug into a syringe and placing on the mucosal atomizer, which are remarkably more steps than with rectal application. This could explain why it is more difficult to prepare the intranasal MDZ administration and, therefore, rectal applications are used more frequently. However, atomization of a liquid drug with a spray device provides the advantage of increased and faster absorption. In addition, when administered as a mist, the drug is less likely to leak from the nasal cavities compared to its liquid form (16). In veterinary medicine, there are no comparable devices that are species- and breed-specific. Using a device which already contains the MDZ solution and allows precise dosing as well as rapid and optimized nasal application, intranasal administration of rescue medications may lead to increased acceptance and success rates (17). In the current survey, there was no remarkable difference in owner-reported success rates between the use of the MAD and a normal syringe, however this can only be reliable assessed in clinical trials. In addition, even though MDZ is buffered to an irritant pH solution, which may increase the risk of transient nasal irritation, the cost–benefit balance can favour its use in the treatment of life-threatening seizure emergencies (24). Lastly, MDZ is more expensive than DZP, at least in some European countries (25), which could be another explanation for the lower rate of use. Based on the results of this survey, DZP was commonly used because of its reduced cost compared to MDZ.

The administration of BZDs is generally considered a crucial measure for the emergency treatment of epileptic seizures; however, based on the results of this survey, this is not always the first choice of action. Many owners retrieved veterinary help as their first option when dealing with seizure emergencies rather than immediately administering rescue medication. A large percentage of owners who seek veterinary help decide to do so in the first 5 minutes after seizure onset. It can be speculated that the owners either did not administer emergency treatment or did not allow adequate time for the rescue medication to act before seeking veterinary assistance. This might be related to the fact that the owners underestimate the enormous benefits of quick intervention and early seizure cessation, doubt the efficacy of rescue medications, lack any form of rescue medication access at home, or have fearful emotions preventing them taking any action at home. With the aim to address this situation and reduce the owners’ reluctance to administer rescue medication, thorough education and training of the owners on how to treat seizure emergencies at home is by far vital. Such an initiative from veterinarians will lead to early management and potential termination of the seizures emergencies, which might prevent patient hospitalisation, development of refractory SE stages, occurrence of serious complications, and increase in the mortality rates.

There are obvious limitations to the study. The main constraint of this research stems from the inherent questionnaire-based study design, introducing bias in evaluating the efficacy of various emergency medications and their choice. Nevertheless, the owners’ perception and opinion are important for treatment success and needs to be considered, when developing better acute seizure treatments.

## 5. Conclusion

Early administration of potent and rapid-acting rescue medications at home by the owners can lead to multiple benefits including increased probability of seizure termination, prevention of hospitalisation and decrease mortality rates related to refractory stages of SE. The practices used for the management of emergency seizures at home vary among owners and countries. Overall, intranasal MDZ showed better success rates and owner compliance rates, earlier seizure termination time points and required less frequent repeated doses compared to rectal DZP. However, application and preparation of intranasal MDZ was rated more difficult than rectal DZP. This could be addressed by developing ready-to-use devices adapted for veterinary patients or appropriate training of the owners by veterinarians.

## Data availability statement

The raw data supporting the conclusions of this article will be made available by the authors, without undue reservation.

## Author contributions

CK: Conceptualization, Data curation, Formal analysis, Investigation, Writing – original draft. SB: Writing – review & editing. SM: Writing – review & editing. NM: Writing – review & editing. HV: Conceptualization, Methodology, Supervision, Writing – review & editing. MC: Conceptualization, Methodology, Supervision, Writing – review & editing.

## Funding

The author(s) declare financial support was received for the research, authorship, and/or publication of this article. The Deutsche Forschungsgemeinschaft (DFG, German Research Foundation) - 491094227 “Open Access Publication Funding” and the University of Veterinary Medicine Hannover, Foundation for funding the open access manuscript.

## Conflict of interest

The authors declare that the research was conducted in the absence of any commercial or financial relationships that could be construed as a potential conflict of interest.

The author(s) declared that they were an editorial board member of Frontiers, at the time of submission. This had no impact on the peer review process and the final decision.

## Publisher’s note

All claims expressed in this article are solely those of the authors and do not necessarily represent those of their affiliated organizations, or those of the publisher, the editors and the reviewers. Any product that may be evaluated in this article, or claim that may be made by its manufacturer, is not guaranteed or endorsed by the publisher.

## Footnotes

^1^ https://www.limesurvey.org/de/

## Abbreviations

SE: status epilepticus
CS: cluster seizure
DZP: diazepam
MDZ: midazolam
MAD: mucosal atomization device
QoL: Quality of life
